# Phosphorus-Based Flame-Retardant Acrylonitrile Butadiene Styrene Copolymer with Enhanced Mechanical Properties by Combining Ultrahigh Molecular Weight Silicone Rubber and Ethylene Methyl Acrylate Copolymer

**DOI:** 10.3390/polym16070923

**Published:** 2024-03-27

**Authors:** Farnaz Ghonjizade-Samani, Laia Haurie, Ramón Malet, Marc Pérez, Vera Realinho

**Affiliations:** 1 Poly2 Group, Department of Materials Science and Engineering, Escola Superior d’Enginyeries Industrial, Aeroespacial i Audiovisual de Terrassa (ESEIAAT), Universitat Politècnica de Catalunya (UPC BarcelonaTech), C/ de Colom, 11, 08222 Terrassa, Spain; farnaz.ghonjizadehsamani@upc.edu; 2 Elix Polymers, Polígono Industrial, Ctra. de Vilaseca—La Pineda s/n, 43110 Tarragona, Spain; ramon.maletmurillo@elix-polymers.com (R.M.); marc.perez@elix-polymers.com (M.P.); 3 GICITED Group, Department of Architectural Technology, Escola Politècnica Superior d'Edificació de Barcelona (EPSEB), Universitat Politècnica de Catalunya (UPC BarcelonaTech), Av. Dr. Marañon 44-50, 08028 Barcelona, Spain; laia.haurie@upc.edu

**Keywords:** ABS, flame retardants, mechanical–thermal dynamic behavior, impact strength, thermal stability, flammability

## Abstract

The present work proposes to investigate the effect of an ultrahigh molecular weight silicone rubber (UHMW-SR) and two ethylene methyl acrylate copolymers (EMA) with different methyl acrylate (MA) content on the mechanical and fire performance of a fireproof acrylonitrile butadiene styrene copolymer (ABS) composite, with an optimum amount of ammonium polyphosphate (APP) and aluminum diethyl phosphinate (AlPi). ABS formulations with a global flame retardant weight content of 20 wt.% (ABS P) were melt-compounded, with and without EMA and UHMW-SR, in a Brabender mixer. During this batch process, ABS P formulations with UHMW-SR and/or EMA registered lower torque values than those of ABS P. By means of scanning electron microscopy (SEM), it was possible to observe that all ABS composites exhibited a homogenous structure without phase separation or particle agglomeration. Slightly improved interfacial interaction between the well-dispersed flame-retardant particles in the presence of EMA and/or UHMW-SR was also noticed. Furthermore, synergies in mechanical properties by adding both EMA and UHMW-SR into ABS P were ascertained. An enhancement of molecular mobility that contributed to the softening of ABS P was observed under dynamic mechanical thermal analysis (DMTA). An improvement of its flexibility, ductility and toughness were also registered under three-point-bending trials, and even more remarkable synergies were noticed in Charpy notched impact strength. Particularly, a 212% increase was achieved when 5 wt.% of EMA with 29 wt.% of MA and 2 wt.% of UHMW-SR in ABS P (ABS E29 S P) were added. Thermogravimetric analysis (TGA) showed that the presence of EMA copolymers in ABS P formulations did not interfere with its thermal decomposition, whereas UHMW-SR presence decreased its thermal stability at the beginning of the decomposition. Although the addition of EMA or UHMW-SR, as well as the combination of both in ABS P increased the pHRR in cone calorimetry, UL 94 V-0 classification was maintained for all flame-retarded ABS composites. In addition, through SEM analysis of cone calorimetry sample residue, a more cohesive surface char layer, with Si-O-C network formation confirmed by Fourier transform infrared (FTIR), was shown in ABS P formulations with UHMW-SR.

## 1. Introduction

Acrylonitrile butadiene styrene (ABS), an engineering thermoplastic, consisting of polybutadiene as a discrete phase and styrene–acrylonitrile copolymer as a continuous phase has been widely used in many industrial applications due to its toughness, impact resistance, chemical resistance and easy processing [1,2,3]. However, the high flammability of ABS combined with its smoke production limits its applications. In order to find a solution for its inherent high flammability and suppress the production of dark smoke, the use of different flame-retardant additives (FRs) in ABS has been studied [4,5,6]. Most of the drawbacks related to flame retardancy of ABS were considered overcome by introducing brominated FRs into the market [5]. However, in the 1990s, it was reported that some of these FRs, like poly brominated diphenyl ethers (PBDEs), release toxic gasses and adversely affect the environment and human health [7]. Therefore, halogen-free flame retardants (HFFRs), based on phosphorus-containing compounds with different oxidation states were developed and introduced to the industry as more environmentally friendly solutions [8,9,10].

The effect of different phosphorus-based FRs, such as triaryl phosphates, resorcinol bis (diphenyl phosphate) (RDP), bisphenol A bis (diphenyl phosphate) (BDP) and red phosphorus (RP), in ABS blends are well-reported [11,12]. The flame-retardant efficiency of ammonium polyphosphate (APP) with the incorporation of other flame retardants such as pentaerythritol (PER) [13], poly-(4-nitrophenoxy)-phosphazene (DPP) [14], montmorillonite (MMT) [15] and aluminum diethylphosphinate (AlPi) [16] in ABS has been also previously studied. ABS combined with piperazine pyrophosphate (PAPP) and AlPi with a PAPP/AlPi mass ratio of 4:1 achieved an optimum synergy at a 25 wt.% loading, with a UL-94 V-0 rating, a LOI value of 30.8% and also a 75.4% reduction in pHRR compared to the neat ABS. This FR system acted in both the condensed and gas phase by the formation of a cohesive, uniform and thermally stable char layer with an efficient barrier effect and by fuel dilution and flame inhibition of the evolved gasses, respectively [17]. Furthermore, a UL-94 V-0 classification was also reported by combining 12.5 wt.% of APP and 12.5 wt.% of aluminum diethyl phosphinate (AlPi) in ABS [18], the FR system employed in the present study. It was previously discussed that synergistic interactions between APP and AlPi promoted the liberation of phosphorus radicals at a temperature range, which acted like scavengers of those radicals yielded during ABS decomposition, as well as the formation of a more effective protective layer in the condensed phase.

However, the addition of flame retardants in ABS generally has a negative impact on its toughness, which in turn limits its applications. As a result, special treatments, such as incorporation of compatibilizers and/or impact modifiers, have been suggested. The impact strength enhancement of an ABS with an intumescent system composed of APP, melamine and calcium 3-hydroxy-2,2-bis(hydroxymethyl) propyl phosphate at the ratio of 3:1:1 was reported [19]. For that, a polybutadiene-grafted SAN rubber, containing a 70 wt.% of polybutadiene (ABS-R) was added at different loadings of 4–26 wt.% into the mentioned intumescent flame retardant (IFR) ABS. The highest increase in impact strength (115%) was achieved for the composite containing 15 wt.% of ABS-R and related to an improvement in the boundary adhesion between the IFR and ABS matrix. The effect of a styrene-ethylene/butylene-styrene-maleic anhydride graft (SEBS-g-MA) on a recycled polymeric blend of ABS/high impact polystyrene/polystyrene (9:21:70), containing 2–10 wt.% of TPP and sepiolite with a ratio of 1:1 was also investigated [20]. The blends with 8 wt.% of FR showed a V-1 classification in UL-94 and a 29% LOI value. However, the incorporation of FRs resulted in a drastic reduction in impact strength from 66.12 J/M to 23.5 J/m, while adding 2, 5 and 10 wt.% SEBS-g-MA increased it up to 69.4 J/m, 74.5 J/m and 80.4 J/m, respectively, by increasing the elastomeric phase and, subsequently, the resilience property of the blend. Although 2 and 5 wt.% SEBS-g-MA addition increased the tensile strength from 21.6 MPa to 28.6 MPa and 27.4 MPa, respectively, when 10 wt.% of SEBS-g-MA was added, a lower tensile strength of 25.5 MPa was registered due to the presence of a higher rubbery content where the elastomeric phase started dominating the blend. Therefore, the blend with 5 wt.% of SEBS-g-MA was chosen as the optimized one. It was also reported that the addition of methacrylate butadiene styrene (MBS) and ethylene vinyl acetate (EVA) up to 3 phr significantly increased the impact strength of the polycarbonate (PC)/ABS containing a 10 and 15 phr flame retardant [21]. In general, compatibilizers and impact modifiers act by reducing the crack growth rate and increasing the energy absorbed in the plastic region, which is also called the stress whitened zone [22].

Moreover, a combination of an ethylene methylacrylate copolymer (EMA) and a silicone/poly(n-butyl acrylate) with a styrene-acrylonitrile-maleic anhydride terpolymer shell (Si-MAH) was used as impact modifiers in glass fiber (GF)-reinforced PC composites [23]. It was seen that by increasing EMA content, the bending strength and modulus continuously decreased due to the enhanced mobility of the matrix molecules induced by the EMA elastomer, showing the optimal mechanical properties when 2 wt.% was added. In fact, for this EMA wt.% content, a 39.7% impact strength increase was noticed. In case of Si-MAH addition, the same trend in mechanical behavior was also observed. In this case, by incorporating 2 wt.% of the Si-MAH, a more significant enhancement of impact strength (152%) was registered due to the presence of a silicone-poly(n-butyl acrylate) core with high toughness and the good compatibility between the MAH shell and PC. Nevertheless, at 4–6 wt.% of Si-MAH, large diameter rubber granules appeared, which could contribute to the descending of the uniform contribution and energy absorbing capability of the additive.

With all these considerations in mind, the present work proposes to investigate, for the first time, the effect of combining EMA copolymers with different MA wt.% and an ultrahigh molecular weight polydimethylsiloxane (UHMW-SR) on thermal stability, fire and mechanical behavior of a fireproofing intumescent flame-retardant ABS composite (ABS P), with the aim of developing new ABS formulations with improved fire behavior and tailor-made mechanical properties.

## 2. Materials and Methods

Acrylonitrile butadiene styrene copolymer (ABS) was provided by Elix Polymers (Tarragona, Spain). According to the manufacturer, ABS contains 37 wt.% ABS grafted with 13 wt.% SAN (total of 50 wt.% ABS) and 50 wt.% of ABS pellets, and has an impact strength of 24 kJ/m^2^ and a melt volume rate of 20 cm^3^/10 min, measured at 220 °C and 10 kg. Ammonium polyphosphate (APP), Exolit^®^ AP422, was supplied by Clariant Produkte (Sulzbach, Germany), and used as a flame retardant. APP, with chemical formula (NH_4_PO_3_)_n_, possesses a polymerization degree (n) higher than 1000 and a phosphorus and nitrogen content of 31–32 wt.% and 14–15 wt.%, respectively, with an average particle size of 17 µ and a density of 1.90 g/cm^3^. Aluminum diethyl phosphinate salt (AlPi), OP1230, with the chemical formula of ((C_2_H_5_)_2_PO_2_)_3_Al, was also supplied by Clariant Produkte with a phosphorus content of 23.3–24 wt.% and a density of 1.35 g/cm^3^; an average particle a size of 20–40 µ was reported by the manufacturer. Ethylene methyl acrylate (EMA) with different methyl acrylate (MA) content, LOTRYL^®^ 24MA07T (E24) with an average content of 23–26 wt.% MA and LOTRYL^®^ 29MA03T (E29) with an average content of 27–31 wt.% MA, were provided by Arkema (Colombes, France). The highest available MA content was chosen with the aim of obtaining the optimum impact strength. Silicone rubber (SR), GENIOPLAST^®^ PELLET S, a pelletized silicone gum formulation with a 70 wt.% of ultrahigh molecular weight polydimethylsiloxane and 30 wt.% of fumed silica was supplied by Wacker (Munich, Germany).

All samples were melt-compounded in a mixing chamber (Brabender, Duisburg, Germany) at a bulk temperature of 180 °C with a rotating rate of 60 rpm for 10 min. Previous to melt-compounding, ABS pellets were dried under vacuum at 80 °C for 2 h, with APP and AlPi at 100 °C for 12 h. After compounding, circular plates with a diameter of 75 mm and a thickness of 4 mm and square plates of 120 mm side length and 3.2 mm thickness were prepared by using compression molding techniques in a hot-plate press IQAP-LAP PL-15 (IQAP Masterbatch group S.L., Barcelona, Spain), applying a temperature of 170 °C and 80 bar of pressure. The composition of the samples is listed in Table 1. The flame-retardant system (P) consisted of AlPi and APP with the ratio of 1:1 and an overall content of 20 wt.%.

The microstructure of ABS composites and residue after cone calorimetry were analyzed using a JSM-5610 scanning electron microscope (JEOL, Tokyo, Japan). ABS composites were cryogenically fractured, and all samples were prepared by sputter depositing a thin layer of gold onto their surface in an argon atmosphere using a SCD005 Sputter Coater (Bal-Tec, Los Angeles, CA, USA).

Dynamic mechanical thermal analysis (DMTA) was used to study the viscoelastic response (storage modulus, loss modulus and tan δ) of the samples. A DMA Q800 (TA Instruments, New Castle, DE, USA) was used and calibrated in a dual cantilever configuration. The experiments were performed from 25 °C to 150 °C at a constant heating rate of 2 °C/min and frequency of 1 Hz, applying a dynamic strain of 0.1%. Specimens were cut with an average length of 35 ± 0.1 mm, width of 12.75 ± 0.1 mm and thickness of 3.2 ± 0.1 mm.

Flexural test was conducted according to ASTM D 790. Specimens of 80 × 10 × 3.2 mm^3^ were loaded in three-point bending with a recommended span (L)-to-depth (d) ratio of 16:1 (L = 16 d). The test was conducted on MTS 810 (Material Test System, Eden Prairie, MN, USA) using version 4 of data acquisition software Test Works-II using a load cell of 10 kN at 1 mm/min rate of loading. For each formulation, three to five specimens were tested and an average result was obtained. Flexural strength (σ_f_) was calculated according to a maximum of σ_f_ = (3 PL)/(2 bd^2^), where σ_f_ (MPa) is the stress; P (N) is the load; L is the span (mm); b is the width of beam tested (mm); and d is the depth of beam tested (mm). Flexural strain is given by ε_f_ = (600 sd)/(L^2^), where s is the deflection. The flexural modulus was calculated from the slope of the initial portion of the stress–strain curve.

Charpy notched impact strength was determined using a Zwick HIT 5.5P testing machine (ZwickRoell, Ulm, Germany). Specimens were tested according to ISO 179 standard [24]. Specimens were 70 ± 0.5 mm length, 10 ± 0.1 mm wide and 4 ± 0.1 mm thick and with a notch depth of 2 mm. All specimens were tested using a pendulum size of 1 J at room temperature.

Thermogravimetric analysis (TGA) under a nitrogen atmosphere, using a SMP/PF7548/MET/400W (Mettler Toledo STAR System, Greifensee, Switzerland) with a constant heating rate of 10 °C/min from 30 °C to 800 °C, was performed. For each experiment a mass of 20 mg ± 0.5 mg and a gas flow rate of 100 mL/min were used.

The flammability behavior of flame-retarded ABS composites was analyzed according to the UL-94 standard [25] (Underwriters Laboratories, Evanston, IL, USA) under vertical burning test conditions on 125 × 12.5 × 3.2 mm^3^ specimens. This analysis was also complemented with limiting oxygen index (LOI) measurements following the ISO 4589 standard [26] procedure, on 80 × 10 × 4 mm^3^ specimens.

Reaction to fire tests were carried out by means of a cone calorimeter (INELTEC, Barcelona, Spain) according to ISO 5660 standard [27] procedure. Specimens of ABS and ABS composites with a diameter of 75 ± 0.1 mm and thickness of 4 ± 0.1 mm were irradiated with a constant heat flux of 35 kW/m^2^ using a constant distance between the electrical resistance and the specimen of 25 mm. Heat release rate (HRR) vs. time curves were registered during the tests. Typical fire-reaction parameters such as time to ignition (TTI), peak of the heat release rate (pHRR), the time to PHRR (tPHRR), total heat emitted (THE), effective heat of combustion (EHC), maximum average rate of heat emission (MARHE) and residue were obtained from the cone calorimeter tests.

Fourier transform infrared (FTIR) spectrometer, Nicolet™ 510 (Thermo Fisher Scientific, Waltham, MA, USA), was used to analyze the chemical nature of ABS composites’ residue after fire-reaction test. The samples were prepared by mixing 1 mg of dried samples with 100 mg of pure KBr, pressed at a pressure of 10 Ton into pellets and analyzed using version 9 of OMNIC™ software. Measurements were obtained in the spectral range of 4000 cm^−1^ to 400 cm^−1^.

## 3. Results and Discussion

### 3.1. Melt-Blending Behavior

**Figure 1:**
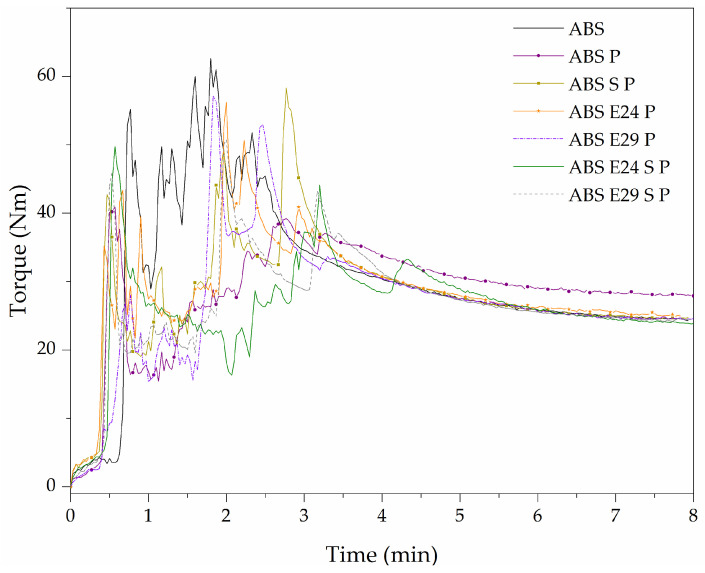
Effect of UHMW-SR and EMA on ABS P torque vs. time of melt-compounding.

Figure 1 presents the variation in torque values versus time for the ABS, ABS P and ABS P with EMA or/and UHMW-SR at 180 °C. The maximum torque and stabilized torque values are listed in Table 2. It should be mentioned that adding EMA and UHMW-SR to the neat ABS did not exhibit significant differences in terms of torque values during the melt-compounding. In Figure 1, as it can be seen, during the first 3 min of blending, the torque values suddenly increased up to a maximum due to material loading, after adding all the materials into the mixing chamber and their consequent melt-melting, the torque values dropped gradually to an equilibrium value. As indicated, a higher torque equilibrium was obtained for the blend samples containing the flame-retardant micro-sized particles. The lowest torque values, among ABS P formulations, were obtained for the blends with EMA and UHMW-SR, which can be due to an improved interfacial interaction between the FR particles and polymeric matrix together with a plasticizing effect of EMA and UHMW-SR that prevented particle agglomeration and reduced the torque equilibrium values.

### 3.2. Morphology of ABS Composites

**Figure 2:**
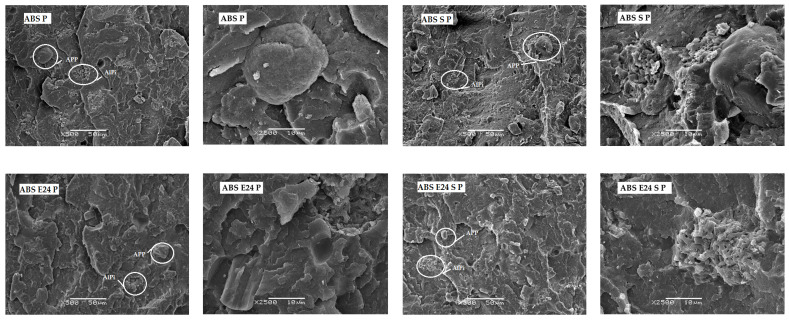

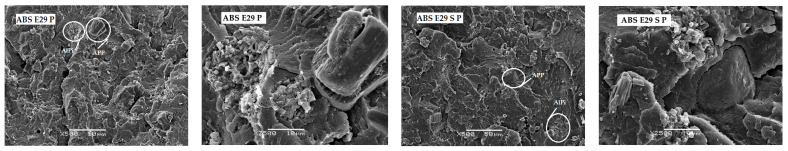
SEM micrographs of ABS P, ABS S P, ABS E24 P, ABS E29 P, ABS E24 S P and ABS E29 S P, at 500× and 2500×, with a scale bar of 50 and 10 µm, respectively.

Figure 2 shows a comparison of the most representative ABS P formulation SEM micrographs. The composites showed a homogeneous structure without any phase separation or particle agglomeration. Also, from these SEM micrographs, it can be seen that AlPi particles were broken during the cryogenic fracture in contrast to APP particles. Examples of APP and AlPi particles were identified within ovals in Figure 2. In addition, by observing the interface of particles and the ABS, it can be seen that by applying EMA or UHMW-SR, the inorganic flame-retardant particles were slightly covered and entangled by the polymeric matrix, which could be due to improved interaction between the particles and polymeric matrix. Furthermore, higher matrix roughness due to a higher ability to plastically deform was also observed in the ABS P samples with EMA and UHMW-SR.

### 3.3. Dynamic Mechanical Thermal Behavior

**Figure 3:**
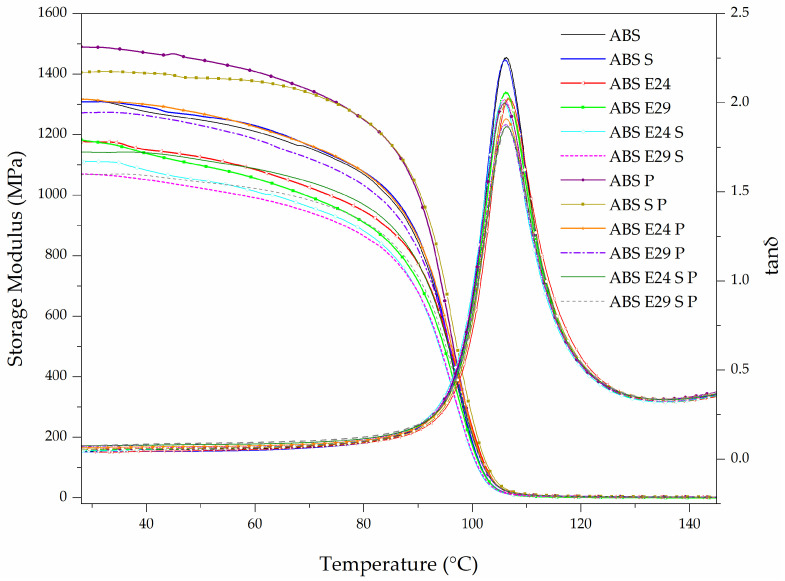
Storage modulus (E′) and tan δ vs. temperature curves of ABS and ABS formulations.

Figure 3 shows the variation in the storage modulus and tan δ with temperature. The samples’ glass transition temperature (Tg) was obtained from the temperature corresponding to the maximum of the tan δ (max tan δ) curves. The storage modulus (E′) was reported from the DMTA curves at 30 °C (see Table 3). As the temperature increased, the storage modulus gradually decreased from room temperature to 80 °C, followed by a sudden decrease, until reaching the energy of free movement of the SAN chain segments at approximately 106 °C. There was an increase in storage modulus values of the ABS P when compared with neat ABS. This observation can be attributed to the hydrodynamic effect of the flame-retardant particles due to the stiffness of these particles as a rigid phase, which cannot be deformed [28]. The use of EMA and UHMW-SR resulted in a continuous decrease in storage modulus and in the intensity of tan δ, indicating an enhancement of mobility of ABS molecules. In Table 3, it can be seen that the efficiency of the additive effect in decreasing the storage modulus was as follows: UHMW-SR < E24 < E29 < UHMW-SR + E24 < UHMW-SR + E29. Also, a small difference between ABS and ABS P storage modules with EMA and UHMW-SR was noticed (Table 3), indicating a major contribution of these elastomers than FR particles to the ABS mechanical behavior.

**Figure 4:**
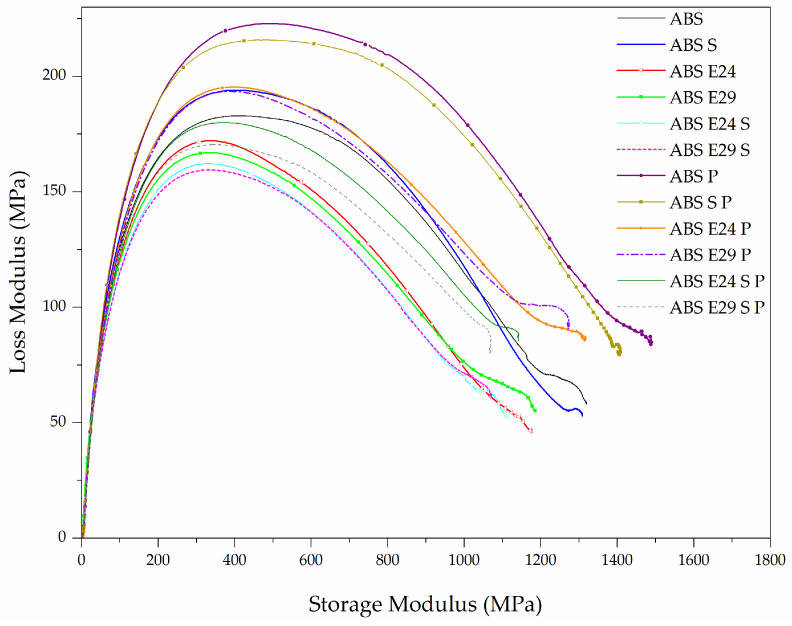
Cole–Cole plots of different samples.

The method developed by Cole–Cole representing the relationship between the real and the imaginary parts of the complex viscosity, respectively (E′, E″) allows the validation of the polymer blend compatibility. Indeed, by plotting loss modulus (E″) values in function of the storage modulus (E′) ones, the compatibility of the blends can be evaluated by observing the shape of the obtained curves. If the blend is readily miscible and homogeneous, the curves are quite smooth and have the shape of a semicircle while the inconsistency of a material system yields a noncircular and irregular graph [29,30]. Figure 4 presents plots of E″ versus E′ for the different formulations. As seen in this figure, the curves of all the formulations present a shape of a semicircle, implying that no phase separation occurred. When both EMA and UHMW-SR were presented in the composites, a smaller radius was observed, which is evidence of compatibility improvement. It can be interpreted that EMA provided polar and nonpolar groups, ethylene (nonpolar) and methyl acrylate (polar), on the ABS chains in the presence of intrinsically hydrophobic UHMW-SR, that led to the reduction in the interfacial tension and made interactions between the polar flame-retardant particles and the polymer matrix stronger, resulting in a more homogeneous composite structure [31].

### 3.4. Flexural Behavior

**Figure 5:**
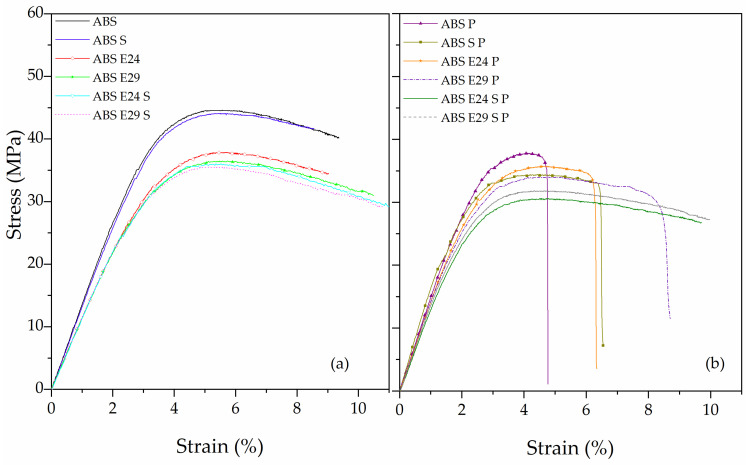
Stress–strain curves of (**a**) ABS and ABS with EMA and/or UHMW-SR, and (**b**) ABS P and ABS P with EMA and/or UHMW-SR, obtained from flexural test.

The flexural behavior of the ABS and ABS composites with EMA and UHMW-SR are shown in Figure 5a. By adding EMA and UHMW-SR to neat ABS, both flexural modulus and strength decreased, resulting in a higher softening effect and improved plastic mechanisms derived by the presence of these elastomeric phases in ABS matrix (see Table 4). All these formulations did not break and showed ductile behavior with significant plastic deformation.

Figure 5b presents strain–stress curves of flame-retardant ABS composite. ABS P showed a quasi-brittle-like fracture behavior with the highest stiffness and lowest plastic deformation before breaking, attributed to the presence of the inorganic stiffer particles. On the other hand, when a small amount of ABS was replaced by EMA copolymer or UHMW-SR, a change in the mode of failure from fragile to ductile, a decrease in maximum flexural strength and an increase in the strain at break were observed. However, when both EMA and UHMW-SR were present, no failure occurred. These samples, ABS E24 S P and ABS E29 S P, showed the highest flexibility, ductility and toughness, indicating an important role of combining EMA and UHMW-SR on the mechanical behavior of ABS and ABS P, in accordance with previous results.

### 3.5. Notched Impact Strength

The effect of combining EMA and UHMW-SR with ABS and ABS P composite on notched Charpy impact strength was investigated. It was observed that incorporating 20 wt.% of flame retardants into the ABS led to a drastic decrease in impact strength to 3.3 kJ/m^2^, in accordance with the results reported in the literature, where by adding different types of halogen-free flame retardants to ABS, a decrease in impact strength from 23 to 2–5 kJ/m^2^ was presented [32]. In Table 5, it can be seen that by adding EMA or UHMW-SR to ABS P, the composite impact strength increased due to the high energy absorption capacity of the flexible molecular chains in EMA and UHMW-SR. In addition, when both EMA and UHMW-SR are added to ABS P, they synergistically improve the impact strength by the highest increase of 164% and 212% for the formulations ABS E24 S P and ABS E29 S P, respectively. It should be mentioned that the effect of E29 on the improvement of impact strength is much higher than E24, which is due to the presence of a higher MA content, the polar part, in E29. To the best of the authors’ knowledge, such a remarkable improvement in the impact strength of a phosphorus-based flame-retarded ABS has not been reported in any published article.

### 3.6. Thermal Stability

**Figure 6:**
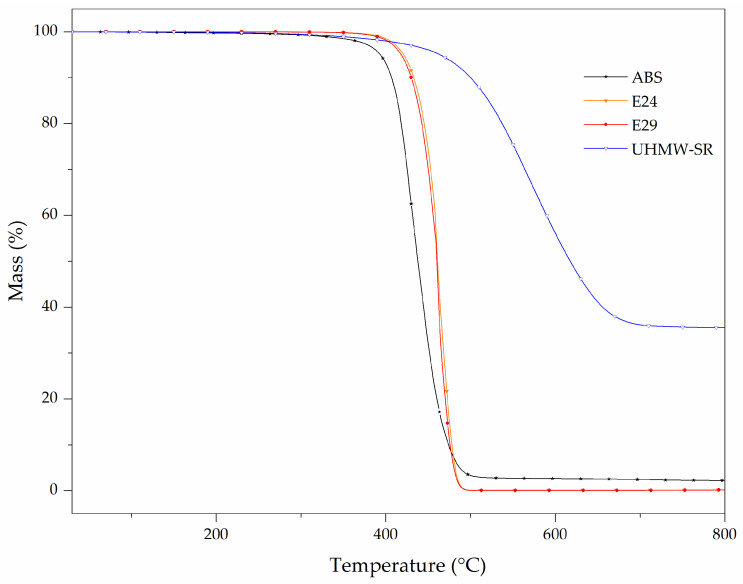
TG curves of E24, E29 and UHMW-SR obtained at 10 °C/min under N_2_ atmosphere.

Mass loss curves of the ABS, E24, E29 and UHMW-SR are shown in Figure 6. Furthermore, the thermal decomposition (TD) steps, the temperature corresponding to 5% of mass loss (T_5%_), maximum mass loss temperature (T_peak_), mass loss (ML) of each TD step and residue at 800 °C are summarized in Table 6.

ABS decomposed in a single step between 260 and 520 °C with a mass loss of 97.8% and a maximum mass loss at 423 °C, leading to a 2.1% residue with no charring effect. It has been previously reported that ABS thermal decomposition starts with the evolution of the butadiene monomer and aromatics from the degradation of the styrenic portion and is followed by the evolution of the acrylonitrile [33,34].

Both EMA copolymers decomposed in a single step between 345 and 540 °C and had similar thermal behavior with no significant residue remaining at 800 °C, indicating that small differences in MA content in EMA do not result in a significant change in its thermal stability under inert atmosphere. Their thermal decomposition is initiated by the homolytic scission of a methoxycarbonyl side group followed by β scission. The loss of the methoxycarbonyl side group has been reported to be the initial decomposition step and scission in the ethylene chain, and played the major role during thermal decomposition [35].

The thermal degradation of UHMW-SR occurred in one main step between 360 and 720 °C with a mass loss of 62.9% (see Table 6), due to the random chain breakage, resulting in a depolymerization of rubber chains and the formation of a small molecular ring structure of cyclic siloxane via rearrangement of silicon–oxygen bonds [36]. Then, the residual Si-OH bonds attacked Si-CH_3_, forming Si-O-Si bonds [37]. At 800 °C, a high amount of 35.8% of residue was registered for UHMW-SR due to the presence of 30 wt.% of silica [38].

**Figure 7:**
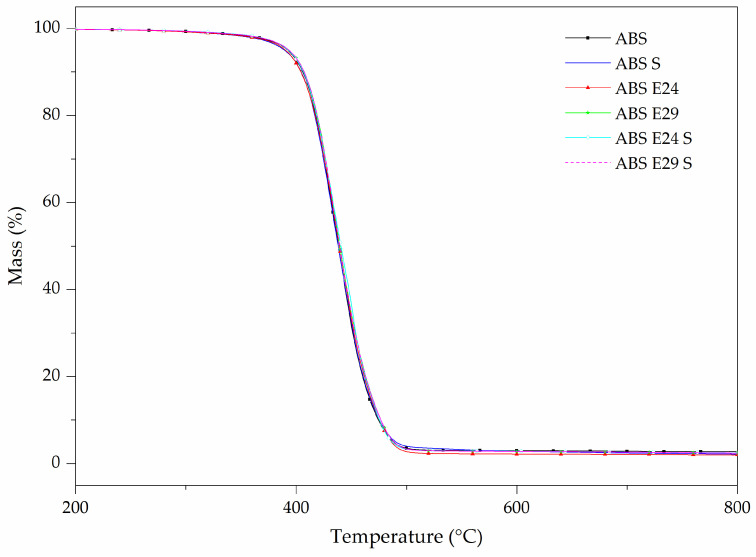
TG curves of ABS with E24 and E29 with or without UHMW-SR.

Furthermore, Figure 7 presents the effect of combining E24 and E29 with or without UHMW-SR in the ABS matrix. The TGA curves of the composites did not show any considerable change in the thermal stability of the composites which can be also due to their small amount incorporated. Similar values of T_5%_ were registered for all samples at 390 °C (see Table 7). However, a slightly higher residue content remained when both EMA and UHMW-SR were presented in the composite compared to their individual addition.

**Figure 8:**
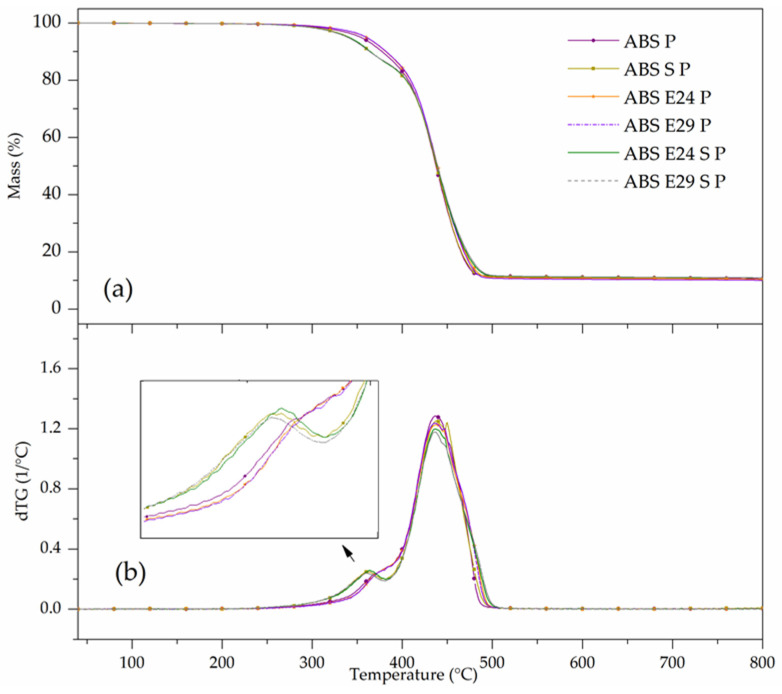
(**a**) TG and (**b**) dTG of curves of flame-retardant ABS formulations.

In Figure 8, by adding 20 wt.% of flame retardants to the ABS matrix, a T_5%_ decrease of 25 °C, as well as a slight increase in the T_peak_ regarding the neat ABS was registered. The presence of flame retardants promoted a 12.6% mass loss between 250 and 390 °C with the maximum mass loss occurring at 365 °C and starting its last decomposition step between 390 °C and 540 °C, resulting in a remaining residue content of 10.5% at 800 °C. By comparing the TGA curves, adding E24 and E29 to the ABS P formulation did not promote any interference in its thermal decomposition behavior; however, by incorporating UHMW-SR, some changes were observed. As it can be seen in Table 8, the presence of UHMW-SR in ABS P showed a T_5%_ decrease of 25 °C, and a slight decrease in the T_peak_ compared to ABS P, registering a 14% of mass loss between 230 °C and 380 °C with the maximum mass loss occurring at 340 °C. When both additives were presented, a slightly higher residue content at 800 °C was registered compared to the ABS P.

### 3.7. Fire Behavior

The flammability behavior of ABS and ABS formulations were analyzed by means of UL-94 vertical burning tests and limiting oxygen index (LOI) measurements. The results of UL-94 tests are summarized in Table 9. As is possible to ascertain, by adding 20 wt.% of flame retardants to ABS, a UL-94 V-0 classification was achieved. Also, adding E24 and E29 with/without UHMW-SR did not affect the mentioned UL-94 classification, being that all flame-retardant ABS formulations remained classified as UL-94 V-0. A similar trend in LOI values when the flame-retardant system was added to the ABS formulations was observed, i.e., all ABS-based polymer formulations presented a constant LOI value equal to 18%, while the respective formulations with the phosphorus system APP/AlPI (1:1) presented a value of 26%. In this sense, it was found that the EMA and/or UHMW-SR copolymers did not affect the UL-94 classification or the LOI values of ABS and ABS P.

**Figure 9:**
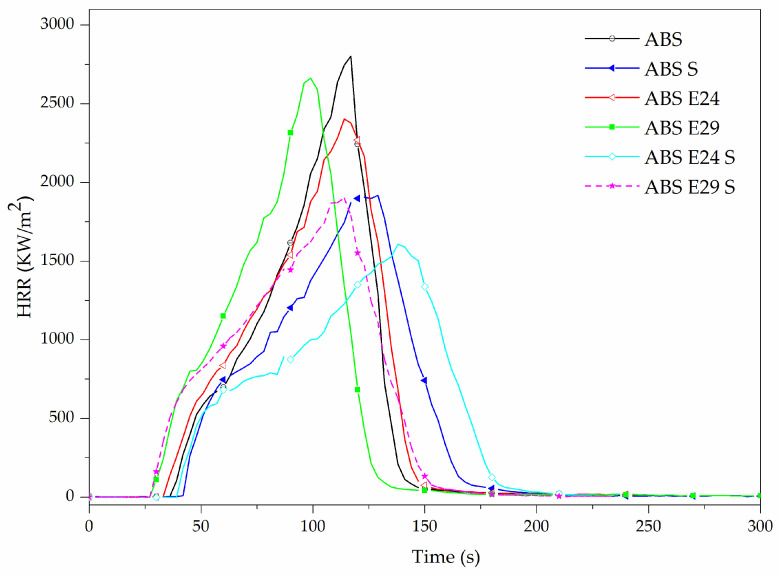
Heat release rate of ABS with either EMA (E24 or E29) copolymers or UHMW-SR and with both of them, (E24/UHMW-SR) or (E29/UHMW-SR).

Furthermore, the heat release rate (HRR) curves for ABS, ABS S, ABS E24, ABS E29 and ABS E29 S, obtained by cone calorimetry test, as a function of time are shown in Figure 9. The heat release rate curve of neat ABS was characteristic of thermally thin samples [39] that elevated quickly after ignition, followed by a less intense increase where the latter increase was related to a growing pyrolysis zone thickness during combustion [40]. Finally, this increase reached its maximum value (2767 kW/m^2^), a sharp peak, with a rapid HRR decrease to the end of burning. This peak behavior was strongly associated with increasing thermal feedback from the back of the sample and with the consumption of the combustible matrix as the result of the burning [18,40]. This feature indicates that the ABS combustion flame spreads rapidly with no remarkable residue (0.26%).

As it can be seen in Table 10, the addition of UHMW-SR to ABS resulted in a 35% and 13% decrease in pHRR and MARHE, respectively, as well as an increase in TTI, exhibiting an improvement in the fire performance of ABS. Furthermore, by comparing the incorporation of EMA with different MA content, it can be noted that for ABS formulations with E24, a lower pHRR and MARHE than E29 were registered. When both UHMW-SR and E24 were present, pHRR was lower compared to their individual addition to ABS. Although, when they were added to ABS P, a different trend was obtained (see Table 11).

**Figure 10:**
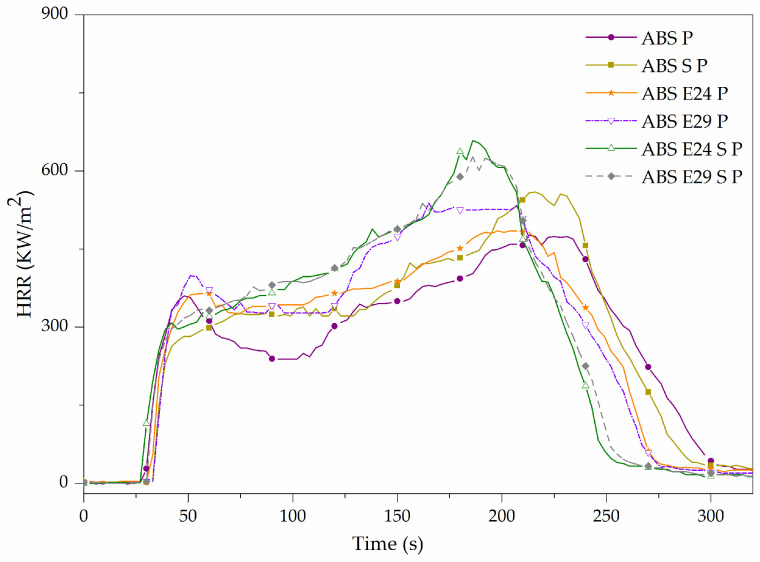
Heat release rate of ABS P formulations.

It was previously reported that the APP/AlPi flame-retardant system of ABS P promotes a combined condensed and gas phase mode of action by the formation of a more effective protective layer and flame inhibition [18]. Due to this, a remarkable decrease in EHC was noticed for ABS P regarding ABS (see Table 11). In Figure 10, it can be noted that adding UHMW-SR or EMA copolymers increased the pHRR observed at the ending of the combustion with respect to ABS P. A slight increase in MARHE index for the mentioned formulations was also registered (see Table 11). Furthermore, TTI was slightly delayed and t_combustion_ occurred earlier, implying that the combustion took place in a shorter period of time compared to ABS P. However, the EHC values of flame-retardant formulations were hardly affected by the presence of EMA and/or UHMW-SR. It should be mentioned that the slightly higher residue formation of ABS P formulations with UHMW-SR could be due to a yield enhancement of crosslinking reactions in the condensed phase that led to a higher char forming ability combined with the presence of silica in this additive.

### 3.8. Morphological and Chemical Analysis of Residue

**Figure 11:**
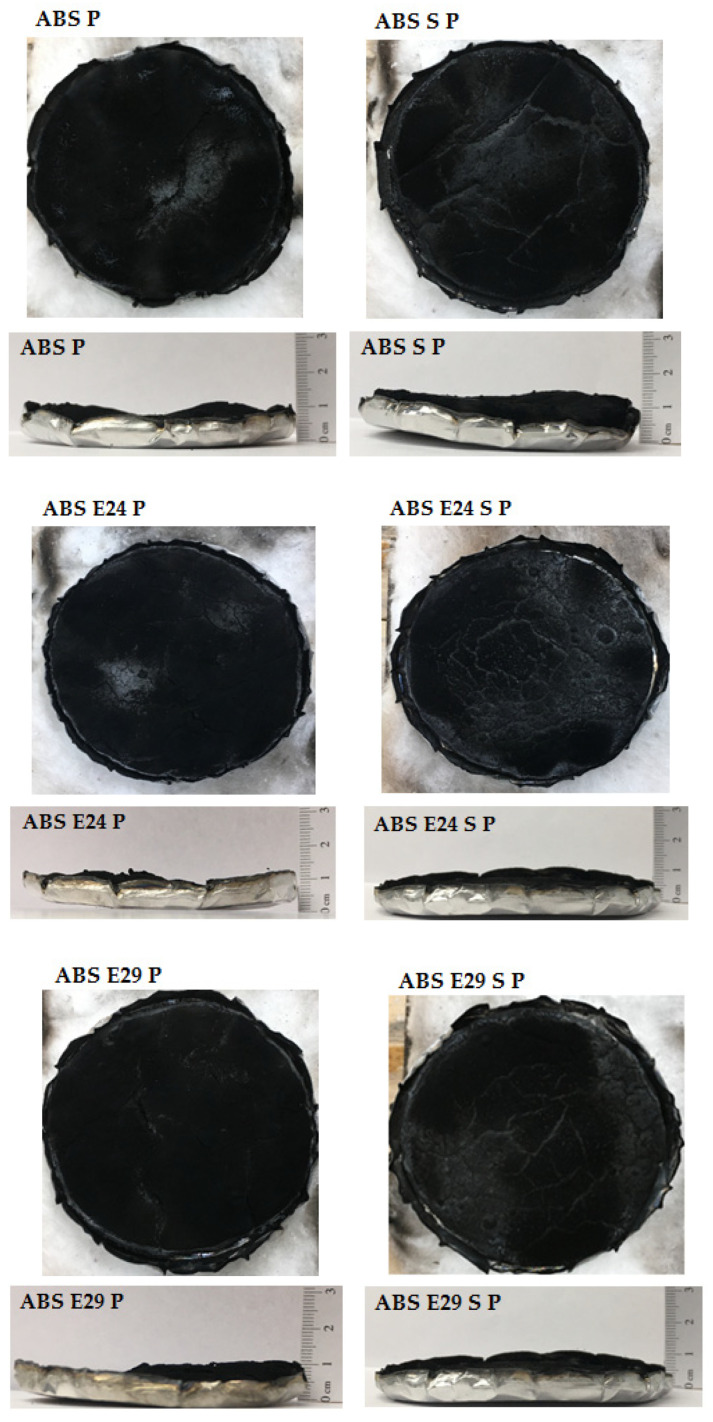
Digital photographs of ABS, ABS S, ABS P, ABS S P, ABS E24 P, ABS E24 S P, ABS E29 P and ABS E29 S P after CC, respectively.

The digital photographs of flame-retardant ABS formulation residue after cone calorimetry are shown in Figure 11. As is possible to observe, all formulations produced a uniform and compact char residue. Nevertheless, by adding UHMW-SR or UHMW-SR and EMA into ABS P, a slightly higher residue expansion degree than that of ABS P was observed.

**Figure 12:**
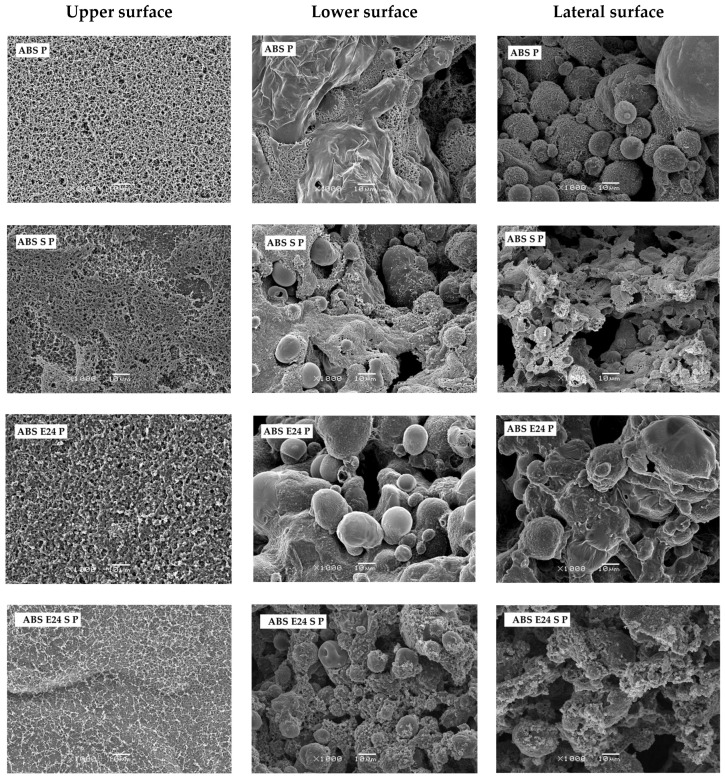

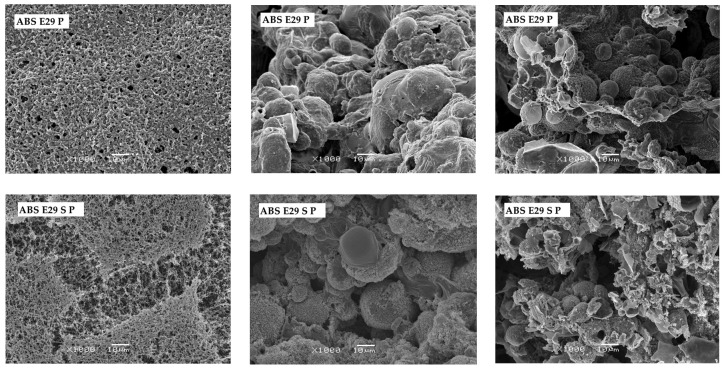
SEM micrographs of the upper (from left), lower and lateral surfaces of the ABS P, ABS S P, ABS E24 P, ABS E24 S P, ABS E29 P and ABS E29 S P after the cone calorimeter tests (at 1000× with a scale bar of 10 µm).

The upper surface and inner structure (lower and lateral surface) of flame-retardant ABS formulation residue after cone calorimetry were observed by SEM (Figure 12). At microscale, ABS S P and ABS E24 S P showed a more cohesive and continuous char layer on the upper surface. Meanwhile, on the remaining formulations, microporosities were observed. Moreover, ABS P showed a foamed inner structure with closed microspheres that acted as an effective physical barrier and heat insulator, in agreement with cone calorimetry results. Although the remaining residue formulations exhibited a similar inner structure to that of ABS P, they showed some cavities which acted like gas escaping channels upon the thermal decomposition of samples, reducing the physical barrier effect of the foamed char layer. During the combustion, this interference on the condensed phase led to an increase in the heat release rate, as previously reported in cone calorimetry results.

**Figure 13:**
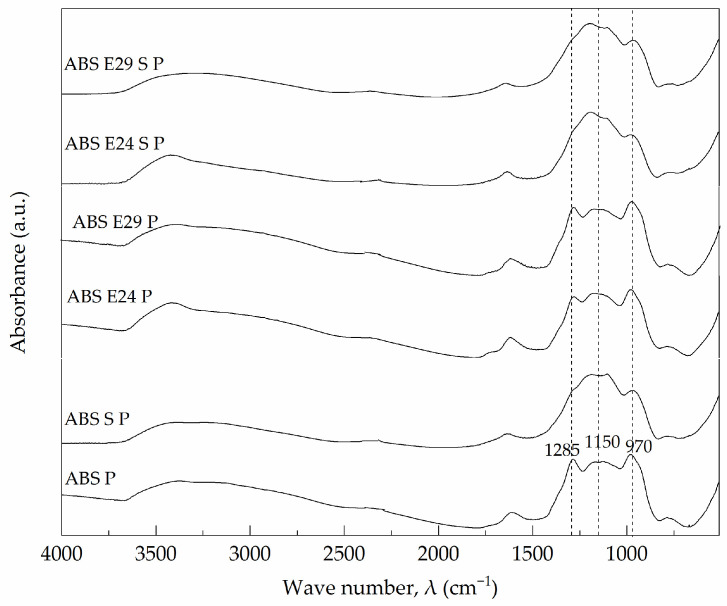
FTIR spectra of flame-retardant ABS residues after cone calorimeter test.

In Figure 13, FTIR spectra of the flame-retarded ABS exhibited the absorbance peaks at 1285 cm^−1^ and 1150 cm^−1^ that were characteristics of P=O and P-O stretching, respectively, due to the formation of the cross-linked ultraphosphates in the char layer, promoted by the dehydration reactions [41,42]. Also, the signal at 970 cm^−1^, attributed to symmetric stretching of the P-O bond in P-O-C structure was registered [43]. It can be noted that when UHMW-SR was presented in the flame-retardant ABS composites, the peaks between 1300 and 1000 cm^−1^ were changed into a single broader band due to the presence of the Si-O-C characteristic band at 1110 cm^−1^ that overlapped them. FTIR spectra of the formulation with E24 or E29, individually, showed no difference in the registered absorbance peaks regarding ABS P.

## 4. Conclusions

In the present study, the effect of UHMW-SR and EMA on mechanical properties and fire behavior of a phosphorus flame-retarded ABS composite containing 10 wt.% APP and 10 wt.% AlPi was investigated.

The lowest equilibrium torque values were obtained for the blends with both EMA and UHMW-SR due to a plasticizing effect and/or an improved interaction between the flame-retardant particles and ABS matrix during the melt blending process. SEM analysis of the fractured surface of the ABS P formulations also showed a homogenous structure without phase separation or particle agglomeration.

The presence of EMA and UHMW-SR in ABS or ABS P led to a decrease in storage modulus and the intensity of tan δ analyzed by means of DMTA. The same trend was also registered for flexural modulus, indicating the effect of EMA and UHMW-SR in the enhancement of molecular mobility leading to a higher flexibility of ABS composites. A synergistic improvement in Charpy notched impact strength was noted when both additives were added. Specifically, the highest impact strength of 10.3 kJ/m^2^, a 212% increase compared to ABS P, was achieved when 5 wt.% of EMA with a MA content of 29% was combined with a 2 wt.% of UHMW-SR in ABS P.

The addition of UHMW-SR to ABS P formulations decreased thermal stability at the beginning of decomposition in TGA; meanwhile, adding EMA copolymers had no effect on its thermal stability. Furthermore, when both EMA and UHMW-SR were present in ABS P, the combustion occurred in a shorter period of time with higher heat release rate values registered in cone calorimetry. However, UL 94 V-0 classification, as well as a LOI value of 26%, was maintained for all flame-retarded ABS formulations, indicating that EMA and UHMW-SR did not promote an adverse effect on their flammability. In addition, SEM analysis of residue after cone calorimetry confirmed the formation of an inner foamed char structure in all ABS flame-retardant formulations. However, some cavities were noticed in ABS P with EMA and/or UHMW-SR. FT-IR analysis showed evidence of the P-O-C cross-linked structure present in all ABS P composites and of Si-O-C in those with UHMW-SR that also showed the highest residue formation in cone calorimetry.

From this study, it has been possible to extend the application window of a self-extinguishing phosphorous flame-retardant ABS, a more environmentally friendly alternative compared to halogen-based ones. Due to the substantial increase in its impact strength by combining an EMA copolymer and an UHMW-SR, as well as maintaining UL-94 V-0 classification, it can be proposed to be used, for instance, in lightweight parts for electronic and electric devices in 5G technology, among others.

## 5. Patents

Regarding this research, a European patent application with the number EP23383250 was filed on 4 December 2023.

## Figures and Tables

**Table 1:** Sample identification and component composition by weight %.

Materials	ABS (wt.%)	P (wt.%)	E24 (wt.%)	E29 (wt.%)	UHMW-SR (wt.%)
ABS	100	-	-	-	-
ABS S	98	-	-	-	2
ABS E24	95	-	5	-	-
ABS E29	95	-	-	5	-
ABS E24 S	93	-	5	-	2
ABS E29 S	93	-	-	5	2
ABS P	80	20	-	-	-
ABS S P	78	20	-	-	2
ABS E24 P	75	20	5	-	-
ABS E29 P	75	20	-	5	-
ABS E24 S P	73	20	5	-	2
ABS E29 S P	73	20	-	5	2

**Table 2:** Torque values, effect of UHMW-SR and EMA on ABS P.

Sample	Average of Torque (Nm)	Max of Torque (Nm)	Stabilized of Torque (Nm)
ABS	30	63	25.5
ABS P	28	48	29.0
ABS S P	26	55	25.5
ABS E24 P	26	50	26.1
ABS E29 P	26	55	25.5
ABS E24 S P	26	53	25.9
ABS E29 S P	26	49	25.3

**Table 3:** Storage modulus (E′), intensity of tan δ and glass transition temperature (T_g_) of ABS and ABS formulations.

Sample	E′ at 30 °C (MPa)	Intensity of tan δ	Tg (°C)
			Max tan δ
ABS	1345 ± 29	2.23 ± 0.03	107 ± 0.4
ABS S	1307 ± 2	2.18 ± 0.05	106 ± 0.2
ABS E24	1176 ± 5	2.01 ± 0.01	107 ± 0.1
ABS E29	1175 ± 4	2.03 ± 0.04	106 ± 0.2
ABS E24 S	1104 ± 10	2.03 ± 0.03	106 ± 0.1
ABS E29 S	1066 ± 3	2.01 ± 0.01	106 ± 0.1
ABS P	1478 ± 15	2.01 ± 0.02	106 ± 0.2
ABS S P	1371 ± 32	2.02 ± 0.01	107 ± 0.1
ABS E24 P	1317 ± 2	1.95 ± 0.03	106 ± 0.1
ABS E29 P	1267 ± 9	1.88 ±0.01	106 ± 0.1
ABS E24 S P	1163 ± 24	1.86 ± 0.01	106 ± 0.1
ABS E29 S P	1067 ± 7	1.86 ± 0.03	107 ± 0.3

**Table 4:** Main flexural properties of ABS and ABS composites.

Sample	Flexural Modulus (GPa)	Maximum Flexural Strength (MPa)	Strain at Maximum Strength (%)	Strain at Break (%)
ABS	1.35 ± 0.02	44.0 ± 0.8	5.5 ± 0.1	NB *
ABS S	1.30 ± 0.02	43.8 ± 0.1	4.8 ± 0.4	NB
ABS E24	1.10 ± 0.02	38.1 ± 0.4	5.7 ± 0.1	NB
ABS E29	1.11 ± 0.01	36.3 ± 0.4	5.4 ± 0.1	NB
ABS E24 S	1.11 ± 0.01	36.5 ± 0.1	5.5 ± 0.2	NB
ABS E29 S	1.12 ± 0.01	36.2 ± 0.9	5.2 ± 0.1	NB
ABS P	1.72 ± 0.05	37.6 ± 3.2	4.1 ± 0.6	4.7 ± 0.4
ABS S P	1.64 ± 0.03	34.4 ± 0.1	4.4 ± 0.2	6.5 ± 0.1
ABS E24 P	1.44 ± 0.01	35.9 ± 0.2	4.8 ± 0.4	6.3 ± 0.3
ABS E29 P	1.44 ± 0.01	33.7 ± 0.5	4.5 ± 0.1	8.5 ± 0.1
ABS E24 S P	1.33 ± 0.01	30.3 ± 0.4	4.7 ± 0.2	NB
ABS E29 S P	1.34 ±0.01	32.4 ±0.8	4.3 ± 0.1	NB

* NB: No break.

**Table 5:** Charpy impact results of ABS P with or without EMA and UHMW-SR.

Samples	Impact Strength (kJ/m^2^)
ABS P	3.3 ± 0.2
ABS S P	5.4 ± 0.1
ABS E24 P	5.9 ± 0.6
ABS E29 P	6.5 ± 0.3
ABS E24 S P	8.7 ± 0.4
ABS E29 S P	10.3 ± 0.4

**Table 6:** TG and dTG data of thermal degradation and residue of E24, E29 and UHMW-SR.

Materials	TD Step	T_5%_ (°C)	T_peak_ (°C)	ML (%)	R_800 °C_ (%)
ABS	1	390	440	96.5	2.1
E24	1	420	460	99.6	0.2
E29	1	420	460	99.7	0.2
UHMW-SR	1	455	580	62.9	35.8

**Table 7:** TG and dTG data of thermal degradation and residue of ABS with E24 and E29 with or without UHMW-SR.

Materials	TD Step	T_5%_ (°C)	T_peak_ (°C)	ML (%)	R_800 °C_ (%)
ABS S	1	390	440	96.3	2.1
ABS E24	1	390	440	97.5	1.9
ABS E29	1	390	440	96.7	2.4
ABS E24 S	1	390	440	96.9	2.2
ABS E29 S	1	390	440	96.7	2.5

**Table 8:** TG and dTG data of thermal degradation and residue of flame-retardant ABS formulations.

Materials	TD Step	T_5%_ (°C)	T_peak_ (°C)	ML (%)	R_800 °C_ (%)
ABS P	1	365	370	12.6	10.5
	2		440	75.1	
ABS S P	1	340	360	13.0	10.7
	2		440	75.5	
ABS E24 P	1	365	370	12.1	10.5
	2		440	76.7	
ABS E29 P	1	365	370	12.2	10.4
	2		440	76.8	
ABS E24 S P	1	340	360	14.0	11.0
	2		438	74.0	
ABS E29 S P	1	340	360	13.2	11.0
	2		435	75.1	

**Table 9:** Results of UL-94 (3.2 mm) and effect of UHMW-SR and EMA on ABS and ABS P.

Samples	UL-94	Dripping	t_1_ + t_2_ (s)
ABS	NC	Yes	-
ABS S	NC	Yes	-
ABS E24	NC	Yes	-
ABS E29	NC	Yes	-
ABS E24 S	NC	Yes	-
ABS E29 S	NC	Yes	-
ABS P	V-0	No	3 + 8
ABS S P	V-0	No	1 + 7
ABS E24 P	V-0	No	2 + 8
ABS E29 P	V-0	No	2 + 4
ABS E24 S P	V-0	No	3 + 7
ABS E29 S P	V-0	No	2 + 3

**Table 10:** Main results obtained from cone calorimeter tests of ABS with or without EMA and UHMW-SR.

Material Code	TTI (s)	pHRR (kW/m^2^)	t_PHRR_ (s)	t _combustion_ (s)	THE (MJ/m^2^)	EHC (MJ/Kg)	MARHE (kW/m^2^)	Residue (wt.%)
ABS	35 ± 1	2767 ± 210	118 ± 2	142 ± 8	133 ± 9	32 ± 2	909 ± 51	0.26 ± 0.07
ABS S	40 ± 7	1815 ± 144	132 ± 4	174 ± 3	129 ± 10	32 ± 2	786 ± 45	1.02 ± 0.03
ABS E24	32 ± 1	2379 ± 190	116 ± 2	135 ± 7	143 ± 1	35 ± 1	1037 ± 26	0.42 ± 0.03
ABS E29	29 ± 3	2535 ± 179	108 ± 5	129 ± 6	133 ± 7	34 ± 2	1046 ± 52	0.24 ± 0.02
ABS E24 S	36 ± 5	1715 ± 153	135 ± 4	187 ± 3	134 ± 2	33 ± 1	765 ± 39	0.95 ± 0.05
ABS E29 S	29 ± 1	1747 ± 116	120 ± 8	156 ± 8	130 ± 6	33 ± 2	848 ± 27	0.93 ± 0.01

**Table 11:** Main results obtained from cone calorimeter tests for flame-retarded ABS.

Materials	TTI (s)	pHRR (kW/m^2^)	t_PHRR_ (s)	t _combustion_ (s)	THE (MJ/m^2^)	EHC (MJ/Kg)	MARHE (kW/m^2^)	Residue (wt.%)
ABS P	24 ± 1	496 ± 27	209 ± 6	305 ± 8	91 ± 8	23 ± 2	336 ± 19	9.04 ± 0.46
ABS S P	27 ± 4	559 ± 1	211 ± 4	288 ± 16	94 ± 1	23 ± 1	338 ± 4	10.26 ± 0.15
ABS E24 P	30 ± 1	514 ± 42	189 ± 10	249 ± 15	93 ± 2	23 ± 1	350 ± 17	8.35 ± 0.17
ABS E29 P	28 ± 2	529 ± 13	177 ± 17	269 ± 8	94 ± 1	24 ± 1	364 ± 12	8.40 ± 0.01
ABS E24 S P	27 ± 1	643 ± 20	186 ± 1	264 ± 1	93 ± 1	24 ± 1	376 ± 1	10.23 ± 0.09
ABS E29 S P	29 ± 1	627 ± 1	189 ± 4	283 ± 4	94 ± 2	24 ± 1	372 ± 5	10.18 ± 0.01

## Data Availability

Data are contained within the article.
